# Phytochemical Analysis and Cholinesterase Inhibitory and Antioxidant Activities of *Enhydra fluctuans* Relevant in the Management of Alzheimer's Disease

**DOI:** 10.1155/2021/8862025

**Published:** 2021-01-11

**Authors:** Simin Shabnam Lopa, Md. Yusuf Al-Amin, Md. Kamrul Hasan, Md. Salim Ahammed, KM Monirul Islam, A. H. M. Khurshid Alam, Toshihisa Tanaka, Md. Golam Sadik

**Affiliations:** ^1^Department of Pharmacy, University of Rajshahi, Rajshahi-6205, Bangladesh; ^2^Department of Psychiatry, Osaka University Graduate School of Medicine, Osaka, Japan

## Abstract

*Enhydra fluctuans*, a popular vegetable in Bangladesh, is used in folk medicine to treat diseases of the nervous system. The objective of this study was to investigate the phytochemical profile and cholinesterase inhibitory and antioxidant potential of the extracts of *E*. *fluctuans*. Among the four tested extracts, the chloroform extract was found to exert the highest inhibition against both the acetylcholinesterase and butyrylcholinesterase enzymes with the IC_50_ (concentration required for 50% inhibition) values of 83.90 *μ*g/mL and 48.14 *μ*g/mL, respectively. Likewise, the chloroform extract showed the highest radical scavenging activity and reducing power. In DPPH radical scavenging assay, the IC_50_ value was found to be 113.27 *μ*g/mL, and in reducing power assay, the absorbance was found to be 1.916 at a concentration of 50 *μ*g/mL. Phytochemical analyses revealed that the chloroform extract contained 19.16 mg gallic acid equivalent (GAE)/g extract of phenolics and 41.84 mg catechin equivalent (CE)/g extract of flavonoids, which appeared to be the highest among the extracts. A significant correlation was observed between phenolic content and butyrylcholinesterase inhibition and antioxidant activity, while a moderate correlation was seen between flavonoid content and cholinesterase inhibition and antioxidant activity. These findings suggest that *E*. *fluctuans* is a natural source of cholinesterase inhibitors and antioxidants, which could be utilized as functional foods for Alzheimer's disease management.

## 1. Introduction

Alzheimer's disease (AD), identified in 1907, is the still most devastating degenerative disorder of the elderly people that damages the brain and results in loss of memory and cognition. In 2019, the number of AD patients was estimated to be 50 million worldwide, which is expected to be tripled by 2050 [1]. One of the major reasons for the increased prevalence of AD is the lack of effective drugs for its complete cure. The development of potent AD drugs is therefore a great challenge to control this devastating disorder. Currently, the most promising approach to prevent and treat AD is to increase the level of acetylcholine in the brain, which gradually becomes deficient during the course of the disease, using cholinesterase inhibitors [2]. Inhibitors of cholinesterases, the enzymes that catalyze the breakdown of acetylcholine, increase the endogenous level of acetylcholine by reducing the catalysis and enhance the cholinergic transmission in the brain, leading to improvement of memory and cognition in AD patients [3, 4]. A large body of data supports that oxidative stress is also involved in the pathogenesis, which suggests the therapeutic use of antioxidants to ameliorate the oxidative-induced progression of AD [5–7]. The multifactorial nature of the disease and the toxicities of the currently used drugs necessitate the development of safe and multitargeted drugs from natural sources. Medicinal plants have already proven to be an important source of drugs with diverse biological activities. Recently, natural foods and food-derived components have received much attention, not only because of safety but also for their capacity to modulate the different targets associated with the pathogenesis [8, 9]. Epidemiologic studies reveal that the consumption of a diet rich in phenolics and flavonoid is significantly associated with a decline in the incidence of AD [10]. Therefore, a systematic investigation of natural foods may provide effective drugs for the management of AD.

*Enhydra fluctuans* is an annual herb and belongs to the family Asteraceae. The plant is found throughout Bangladesh. It is a popular vegetable in Bangladesh and a rich source of nutrition including proteins, carbohydrates, vitamins, and minerals [11]. Besides its consumption as food, the plant has been utilized as a traditional remedy in Bangladesh for the treatment of neuralgia and other nervous system disorders. Among other uses, juice of the leaves is used in inflammation, skin disease, and smallpox. It is useful in the torpidity of the liver [12, 13]. The presence of some phytochemicals was reported from this plant; these include flavonoids, saponins, terpenoids, and tannins. Limited biological investigations have been carried on this plant and suggested that the plant has analgesic, antidiabetic, anthelmintic, thrombolytic, and hepatoprotective properties [14]. However, there is no scientific study available to describe the neuroprotective effect relevant to AD treatment. In our continuing search for acetylcholinesterase inhibitors and antioxidants from Bangladeshi plants, we have selected this plant due to ethnopharmacological relevance and investigated the phytochemical profile and cholinesterase inhibitory and antioxidant activities.

## 2. Materials and Methods

### 2.1. Chemicals

Acetylthiocholine iodide, S-butyrylthiocholine iodide, donepezil, galantamine, catechin, gallic acid, 2,2′-diphenyl-1-picrylhydrazyl (DPPH), Folin-Ciocalteu reagent, Tris-HCl, and Triton X-100 were procured from Sigma-Aldrich, India. Gallic acid, aluminum chloride, ammonium molybdate, and potassium ferricyanide were procured from Merck, India. Methanol, ethyl acetate, chloroform, and petroleum ether were from Active Fine Limited, Dhaka, Bangladesh. Unless mentioned, all of the chemicals used in this study were of analytical grade.

### 2.2. Experimental Animals

10 Swiss albino mice (4-6 weeks old) were obtained from the Animal House, Jahangirnagar University, Savar, Dhaka. Animals were housed in the experimental room and given a standard diet. We complied with the international ethical guidelines to deal with the animals, and the procedures were approved by the Institutional Animal, Medical Ethics, Biosafety and Biosecurity Committee (225/230-IAMEBBC/IBSc), Institute of Biological Sciences, University of Rajshahi, Bangladesh.

### 2.3. Plant Materials

The stems and leaves of *E*. *fluctuans* were gathered from the University of Rajshahi campus, Rajshahi, Bangladesh, in the month of March 2018. The plant species was recognized by an adept taxonomist at the Department of Botany, University of Rajshahi, where a voucher specimen (accession no. 359) has been preserved.

### 2.4. Plant Extraction

Fresh stems and leaves of *E*. *fluctuans* were first washed properly, then cut into small pieces, shade-dried, and ground into coarse powder by employing a grinder. The cold extraction method was used for the extraction of dried plant powder (280 g) with methanol. The resulting extract was filtered and concentrated by a rotary evaporator under reduced pressure at 50°C temperature to achieve the crude methanol extract (20.0 g). A definite amount of the resulting methanolic extract was measured, suspended in water, and then partitioned with petroleum ether, chloroform, ethyl acetate, and water by the method as described earlier [15]. All the extracts were preserved in the refrigerator until use.

### 2.5. Phytochemical Analysis

#### 2.5.1. Phytochemical Screening of the Plant Extract

Various phytochemicals including tannins, phenolics, flavonoids, alkaloids, saponins, and phytosterols were determined by performing preliminary qualitative analysis of the extracts in accordance with the methods as described earlier [15].

#### 2.5.2. Estimation of Total Phenolic Content

FCR (Folin-Ciocalteu reagent) was employed for estimating the total phenolic content of various extractives of *E*. *fluctuans* as described earlier [16]. 0.5 mL of plant extract or solution of reference compound at various concentrations was added to 2.5 mL of 10% FCR and 2.5 mL of 7.5% sodium carbonate solution. The resulting solution was kept at 25°C for 20 minutes, and the absorbance was measured at 760 nm. Gallic acid was utilized as a reference standard. The total phenolic content was calculated from the standard gallic acid graph and presented as mg of gallic acid equivalent (GAE)/g of dried extract.

#### 2.5.3. Estimation of Total Flavonoid Content

The aluminum chloride colorimetric method was employed to measure the total flavonoid content of various extracts of *E*. *fluctuans* using catechin as a reference standard [17]. 1.0 mL of the sample was mixed with 3.0 mL of methanol, 0.2 mL of 1 M potassium acetate, 0.2 mL of 10% aluminum chloride, and 5.6 mL of purified water. The resulting mixture was left at room temperature for 30 minutes, and the absorbance was read at 420 nm. The total flavonoid content was determined from the standard catechin graph and expressed as mg of catechin equivalent (CE)/g of dried extract.

### 2.6. Antioxidant Activity

#### 2.6.1. Estimation of Reducing Power (RP)

The reducing ability of the plant extracts of *E*. *fluctuans* was evaluated by the method as described by Oyaizu [18]. The extract or standard solutions (1 mL) were added to 2.5 mL each of potassium buffer (0.2 M) and potassium ferricyanide (1% *w*/*v*) and mixed gently. The reaction mixture was incubated at 50°C for 20 minutes followed by the addition of 2.5 mL of trichloroacetic acid (10% *w*/*v*) solution. Centrifugation (3000 rpm, 10 minutes) of the mixture was performed to get the clear supernatant solution. Supernatant solution (2.5 mL) was added to 0.5 mL of ferric chloride (0.1% *w*/*v*) solution and distilled water (2.5 mL), and the absorbance of the solution was read by a spectrophotometer at 700 nm. For comparison, catechin was taken as a reference standard.

#### 2.6.2. Estimation of DPPH Radical Scavenging Activity

The method outlined by Choi et al. [19] was followed to estimate the DPPH radical scavenging activity of different extractives of *E*. *fluctuans*. After dissolving the samples (various concentrations) and DPPH (0.135 mM) in methanol solution, the test samples (2 mL) were added into DPPH (3 mL) in different test tubes. To eliminate the effect of light on the reaction rate, the mixture was kept in a dark place for 30 minutes at 25°C, and the absorbance was read by a spectrophotometer at 517 nm. The percent scavenging of DPPH free radical activity (%) of different samples was calculated utilizing the following equation:

(1) $$\begin{array}{c} \left[\frac{A_{\text{absorbance of control}}–A_{\text{absorbance of sample}}}{A_{\text{absorbance of control}}}\right]\times 100. \end{array}$$

A graph of percent scavenging of DPPH free radical activity was plotted against the concentration of each plant extract to calculate the IC_50_ value, which is the concentration required to cause 50% scavenging.

### 2.7. Estimation of Anticholinesterase Activity

The cholinesterase inhibitory activity was assessed by the spectrophotometric method of Ellman et al. [20]. As the source of acetylcholinesterase enzymes, mouse brain homogenate was prepared as described earlier [15]. Centrifugation (2000 g, 10 minutes) of EDTA (1 *μ*g/mL)-treated human blood led to the separation of plasma (supernatant), which was utilized as the butyrylcholinesterase enzyme source for determining butyrylcholinesterase inhibitory activity. Spectrophotometric observation of the hydrolysis of acetylthiocholine iodide by acetylcholinesterase was performed as the final act of the assay. 200 *μ*L of enzyme solution was first blended with 500 *μ*L of the test sample and kept for 20 minutes at 37°C. Just after adding Ellman's reaction mixture (3.5 mL; 0.5 mM acetylthiocholine iodide, 1 mM DTNB) in a sodium phosphate buffer (pH 8.0, 50 mM), absorbance (405 nm) was recorded continuously for 5 minutes at a 1-minute interval. A blank reaction was estimated by taking saline in place of the enzyme, and a control reaction was also assessed by substituting the inhibitor with saline. Donepezil was used as a reference standard. The following formula was used to calculate the percentage inhibition of acetylcholinesterase activity:

(2) $$\begin{array}{c} \left[\frac{A_{\text{absorbance of control}}–A_{\text{absorbance of sample}}}{A_{\text{absorbance of control}}}\right]\times 100. \end{array}$$

For butyrylcholinesterase inhibition assay, the same Ellman's method as mentioned above was followed except that the enzyme solution was 50 *μ*L and butyrylthiocholine iodide was used instead of acetylthiocholine iodide. Galantamine was taken as a reference standard. The butyrylcholinesterase inhibitory activity (% inhibition) was calculated utilizing the same formula as mentioned above for acetylcholinesterase activity. The IC_50_ values for each plant extract were calculated from the graph plotted as percent inhibition against concentration using nonlinear regression analysis.

### 2.8. Statistical Analysis

All analyses were carried out in triplicate. Data were expressed as mean ± SD. Statistical and graphical assessments were performed by utilizing GraphPad Prism 8.0.1 and Microsoft Excel 2010 (Roselle, IL, USA). The *t*-test was conducted to identify significant differences (*P* value < 0.05) between the average values. A correlation study was performed using Pearson's correlation test.

## 3. Results and Discussions

Traditional medicine, which generally uses a wide variety of herbs, natural foods, and spices, is practiced in many countries to treat different diseases including AD. Scientific evaluation of this medicine may lead to the development of new drugs or to establish them as alternative medicine. Nutraceuticals such as epigallocatechin, quercetin, luteolin, resveratrol, curcumin, carotenoids, and vitamins present in the fruits or vegetables have been found to improve memory and cognitive function in AD. They have proved to be multipotent therapeutic molecules, which can interfere with most of the pathological processes in AD [8–10]. In Bangladesh, many food plants are used traditionally to cure the diseases of the nervous system, which might have an impact on brain activity; only a few of them have been scientifically investigated. *E*. *fluctuans*, a very popular vegetable in Bangladesh, is used in the management of neuralgia and other neuronal disorders [12, 13]. Herein, we report that *E*. *fluctuans* possesses cholinesterase inhibitory and antioxidant activities relevant to the management of AD.

### 3.1. Phytochemical Analysis

The plant contains a large number of bioactive compounds with diverse chemical properties. In order to determine the chemical nature of the molecules that might contribute to antioxidant and cholinesterase inhibitory activities, the crude methanol extract (percent yield 7.14% *w*/*w*) of *E*. *fluctuans* was partitioned with four solvents of different polarity, namely, petroleum ether, chloroform, ethyl acetate, and water, according to the Kupchan method [21]. The percent yield of the different partionates/extracts was 35%, 27%, 10%, and 8% (*w*/*w*) for the aqueous extract, petroleum ether extract, chloroform extract, and ethyl acetate extract, respectively, indicating that water has the highest capacity to extract the phytochemicals from this plant. This result also suggests the presence of a high proportion of polar compounds in the plant material.

A preliminary phytochemical analysis conducted on the extracts revealed that the plant contains tannins, phenolics and flavonoids, phytosterols, and saponins. Interestingly, all the tested extracts contained phenolics and flavonoids, but higher amounts were found in the chloroform extract (Table 1). Alkaloid was found to be absent in the plant.

The total phenolic content (TPC) and total flavonoid content (TFC) of the extracts from *E. fluctuans* were determined, and the results have been shown in Table 2. The highest amount of phenolics was found in the chloroform extract (19.16 ± 1.06 mg GAE/g dried extract) followed by the ethyl acetate extract (14.06 ± 1.01 mg GAE/g dried extract). The petroleum ether extract and aqueous extract contained a lower amount of phenolics. Similarly, the highest content of flavonoids was found in the chloroform extract (41.84 ± 1.76 mg CE/g dried extract) followed by the petroleum ether extract (24.86 ± 1.39 mg CE/g dried extract). The ethyl acetate extract and aqueous extract contained a comparatively less amount of flavonoids. The observed differences in the total phenolic content and total flavonoid content of the solvent extracts may be attributed to the differences in the polarity of the solvents. However, a decent amount of phenolics and flavonoids present in the chloroform extract indicated that they might contribute to the biological activity.

Phenolics and flavonoids are secondary metabolites that are widespread in fruits and vegetables. These compounds have many proven health benefits. Phenolics exert antioxidant activity by scavenging free radicals. They are reported to prevent oxidative stress associated with AD through antioxidant activity [22, 23]. Flavonoids are also considered potential natural antioxidants with the ability to scavenge free radicals and reactive oxygen species. They contain conjugated ring structures and hydroxyl groups, which are believed to play a functional role in antioxidant activity. Flavonoids from various natural sources exhibit multiple pharmacological activities including inhibition of cholinesterase and aggregation of amyloid-*β* (A*β*) peptide [24]. In this study, the presence of a considerable amount of phenolics and flavonoids in the extracts of *E*. *fluctuans* indicates that they might have potential as therapeutics in AD.

### 3.2. Cholinesterase Inhibitory Activity

**Figure 1:**
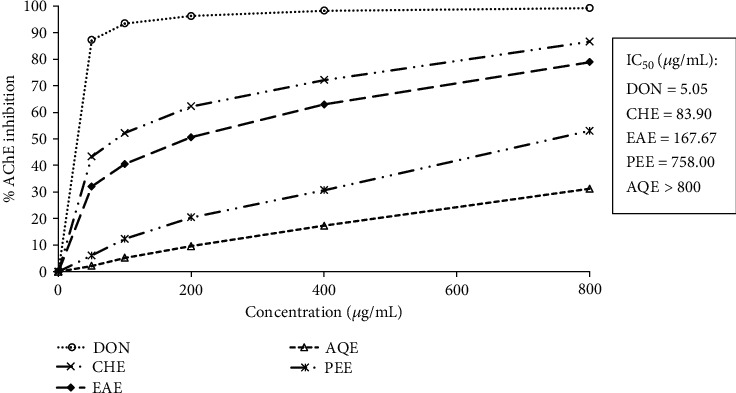
Inhibition of acetylcholinesterase by different extracts of *E*. *fluctuans*. Results represent mean ± SD (*n* = 3). Donepezil (DON) was used as a reference compound. CHE: chloroform extract; EAE: ethyl acetate extract; AQE: aqueous extract; PEE: petroleum ether extract; AChE: acetylcholinesterase.

Because the level of acetylcholine gradually declines in AD, which appeared to be correlated with the impairment of cognition and memory, inhibitors of cholinesterases that elevate the endogenous level of acetylcholine have been accepted as the first-line pharmacotherapeutics for treatment [3, 4]. Acetylcholinesterase is the major cholinesterase in the brain that catalyzes the hydrolysis of acetylcholine and shows higher specificity toward acetylcholine. The *E*. *fluctuans* extracts were examined for inhibition of brain acetylcholinesterase at different concentrations by Ellman's colorimetric method [20]. The results are shown in Figure 1. Along with the test extracts, a reference acetylcholinesterase inhibitor donepezil, which is highly selective for acetylcholinesterase than butyrylcholinesterase [3], was used in this study, and its IC_50_ value was found to be 5.05 *μ*g/mL. The results revealed the acetylcholinesterase inhibitory properties of all four extracts. The highest inhibition was found from the chloroform extract followed by the ethyl acetate extract and petroleum ether extract with the IC_50_ values of 83.90, 167.67, and 758.00 *μ*g/mL, respectively. The aqueous extract had a relatively weak activity (IC_50_ > 800 *μ*g/mL). The activity of the extracts was remarkable when compared with the other medicinal plants such as *Vanda roxburghii*, *Bacopa monnieri*, *Centella asiatica*, *Convolvulus pluricaulis*, and *Aegle marmelos*, which are used traditionally to enhance memory and relevant to AD treatment [15, 22]. Our results suggest that *E*. *fluctuans* possesses an appreciable acetylcholinesterase inhibitory activity.

**Figure 2:**
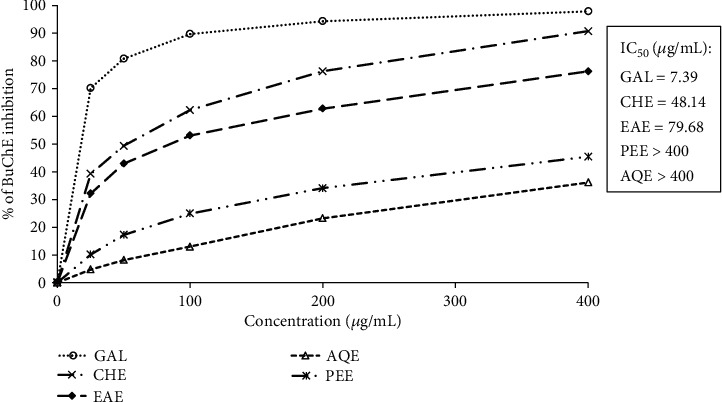
Inhibition of butyrylcholinesterase by different extracts of *E*. *fluctuans*. Results represent mean ± SD (*n* = 3). Galantamine (GAL) was used as a reference compound. CHE: chloroform extract; EAE: ethyl acetate extract; AQE: aqueous extract; PEE: petroleum ether extract; BuChE: butyrylcholinesterase.

Like acetylcholinesterase, butyrylcholinesterase also hydrolyzes the neurotransmitter acetylcholine, although less efficiently, and is hence a viable therapeutic target in AD. Inhibition of butyrylcholinesterase has been found to elevate brain acetylcholine in the brain leading to improvement of memory and cognition. The current AD drugs galantamine and rivastigmine act through inhibition of both acetylcholinesterase and butyrylcholinesterase [25]. We have also assessed the extracts of *E*. *fluctuans* for inhibition of butyrylcholinesterase by the same Ellman's method [20] as employed for acetylcholinesterase. Due to high selectivity for the butyrylcholinesterase enzyme, galantamine was used as the reference butyrylcholinesterase inhibitor [26]. The results have been presented in Figure 2. All the tested extracts inhibited the butyrylcholinesterase enzyme. Similar to the inhibition of acetylcholinesterase, the highest inhibition was found in the chloroform extract followed by the ethyl acetate extract with the IC_50_ values of 48.14 and 79.68 *μ*g/mL, respectively. On the other hand, the petroleum ether extract and aqueous extract exhibited moderate inhibition (IC_50_ values > 400 *μ*g/mL). The lower IC_50_ values of the chloroform and ethyl acetate extracts against butyrylcholinesterase compared with acetylcholinesterase suggest that these extracts have more specificity for butyrylcholinesterase than acetylcholinesterase.

### 3.3. Antioxidant Activity

Oxidative stress is extensive in AD and closely related to the pathogenesis of AD. It is increasingly evident that free radicals are involved in neuronal dysfunction in AD through the peroxidation of membrane lipid. A*β* protein, which is easily aggregated and forms senile plaques, has been shown to be an inducer of free radical production in neuronal cells leading to increased peroxidation of lipid in the brain with AD [5, 6]. Plant extracts or plant-derived molecules such as *Ginkgo biloba*, resveratrol, and curcumin, by virtue of their antioxidant activity, can reduce the oxidative damage to neurons [7–10]. The antioxidant potential of the extracts of *E*. *fluctuans* was evaluated by using free radical scavenging and reducing power *in vitro* models.

**Figure 3:**
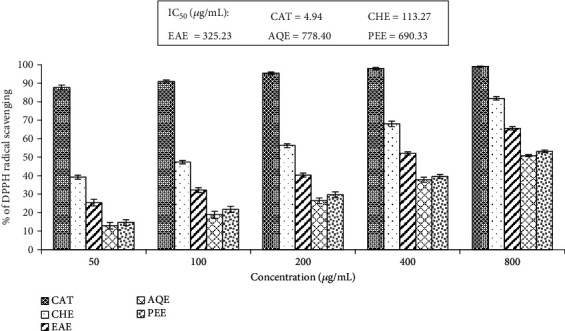
DPPH radical scavenging activity of different extracts of *E*. *fluctuans*. Results represent mean ± SD (*n* = 3). Catechin (CAT) was used as a reference standard. CHE: chloroform extract; EAE: ethyl acetate extract; AQE: aqueous extract; PEE: petroleum ether extract.

DPPH is a molecule containing stable free radicals, whose purple color decreases following actions of proton radical scavengers and which can be monitored by a spectrophotometer. The activity of all the extracts was revealed by the discoloration of DPPH. The percent scavenging of the extracts at different concentrations has been presented in Figure 3. The IC_50_ values of the extracts ranged from 113.27 to 778.40 *μ*g/mL. The highest activity was found in the chloroform extract and the lowest in the aqueous extract. Our results indicated the ability of the extracts of *E*. *fluctuans* to donate an electron or hydrogen, which can react with free radicals.

**Figure 4:**
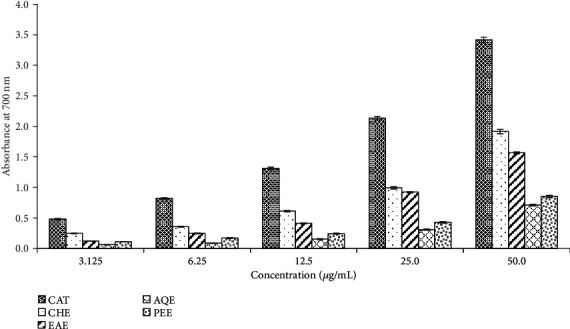
Reducing power of different extracts of *E*. *fluctuans*. Results represent mean ± SD (*n* = 3). Catechin (CAT) was used as a reference standard. CHE: chloroform extract; EAE: ethyl acetate extract; AQE: aqueous extract; PEE: petroleum ether extract.

Reducing power assay, which reflects the capacity of an antioxidant to donate an electron, is another commonly used *in vitro* model to evaluate the antioxidant potential of the plant extracts. In this assay, the reducing capacity of the extracts was determined by their ability to reduce the Fe^3+^-ferricyanide complex to the ferrous form. The reaction could be monitored by the spectrophotometer due to the appearance of the blue color of ferrous ions. The reducing activity of all the extracts was evident by the formation of the color of ferrous ions. The absorbance of the different extracts at different concentrations has been shown in Figure 4. The absorbance of the extract was found to be related to the concentration of the extract. Similar to radical scavenging, the chloroform extract showed the highest reducing activity. The order of potency as judged from the absorbance values was chloroform extract>ethyl acetate extract>petroleum ether extract>aqueous extract. The results of both assays demonstrated the antioxidant potential of *E*. *fluctuans*.

According to the multitargeting approach, many plants based on ethnopharmacological relevance have been investigated to identify the potential candidates having cholinesterase inhibitory property and antioxidant activity. Out of a large number of plants studied, only a few have been reported to exhibit an acceptable level of inhibition of acetylcholinesterase and antioxidant activity [27]. Little information is available regarding dual activity from vegetable sources. This report is the first to show that *E*. *fluctuans* contains an appreciable amount of phenolics and flavonoids and possesses an acceptable level of cholinesterase inhibition and antioxidant activity. These findings validate the traditional use of this plant in central nervous system disorders.

Increasing pieces of evidence suggest a linear relationship between the polyphenolic content and the antioxidant activity of plant materials [28–30]. Phenolics and flavonoids from natural sources have also been found to inhibit cholinesterase enzymes. Thus, correlations were tested between phytochemical contents and cholinesterase inhibitory and antioxidant activity. The results have been presented in Table 3. A significant correlation was observed between phenolic content and butyrylcholinesterase inhibition (*r* = 0.953), DPPH radical scavenging (*r* = 0.999), and reducing power (*r* = 0.999), while a moderate correlation was seen between phenolic content and acetylcholinesterase inhibition (*r* = 0.874) and flavonoid content and cholinesterase inhibition and antioxidant activity (*r* = 0.590-0.674). These results suggest an association of phenolics and flavonoids with the inhibition of cholinesterase as well as antioxidant activity.

## 4. Conclusion

The results obtained in this study demonstrated for the first time that *Enhydra fluctuans* is an important natural source of phenolics and flavonoids with cholinesterase inhibitory and antioxidant activities. This plant can be exploited as functional food/or nutraceutical in the management of AD. The present findings warrant further evaluation of this plant in the experimental models of AD.

## Figures and Tables

**Table 1:** Qualitative phytochemical analysis of different extracts of *E*. *fluctuans*.

Phytoconstituents	CHE	EAE	AQE	PEE
Phenolics	+++	++	+	+
Flavonoids	+++	+	+	++
Alkaloids	-	-	-	
Tannins	-	+	++	-
Phytosterols	+	+	-	++
Saponins	-	+	+	-

+, present in mild amount; ++, present in moderate amount; +++, present in large amount; -, absence. CHE: chloroform extract; EAE: ethyl acetate extract; AQE: aqueous extract; PEE: petroleum ether extract.

**Table 2:** Total phenolic and flavonoid contents of different extracts from *E*. *fluctuans*.

Samples	TPC (mg GAE/g dried extract)	TFC (mg CE/g dried extract)
CHE	19.16 ± 1.06	41.84 ± 1.76
EAE	14.06 ± 1.01	11.82 ± 0.81
AQE	3.03 ± 0.60	3.62 ± 0.43
PEE	4.47 ± 0.80	24.86 ± 1.39

CHE: chloroform extract; EAE: ethyl acetate extract; AQE: aqueous extract; PEE: petroleum ether extract; TPC: total phenolic content; TFC: total flavonoid content; GAE: gallic acid equivalent; CE: catechin equivalent.

**Table 3:** Correlation of total phenolic and flavonoid contents from *E*. *fluctuans* with cholinesterase inhibition and antioxidant activities.

Assays	Correlation coefficient (*r*) values	
	Total phenolic content	Total flavonoid content
DPPH scavenging	0.999	0.680
Reducing power	0.999	0.654
Acetylcholinesterase inhibition	0.874	0.674
Butyrylcholinesterase inhibition	0.953	0.590

## Data Availability

The data used to support the findings of this study are available from the corresponding author upon request.
